# Vitamin D Metabolites in Mother–Infant Dyads and Associated Clinical Outcomes in a Population of Nigerian Women

**DOI:** 10.3390/nu16121857

**Published:** 2024-06-13

**Authors:** Shirley Delair, Ann Anderson-Berry, Eyinade Olateju, Godwin Akaba, Nubwa Medugu, Elizabeth Lyden, Martin Kaufmann, Glenville Jones, Emmanuel Anigilaje, Yunusa Thairu, Nicholas Kocmich, Theresa Ajose, Grace Olanipekun, Amy Rezac-Elgohary, Stephen Obaro, Corrine Hanson

**Affiliations:** 1 Department of Pediatrics, College of Medicine, University of Nebraska Medical Center, Omaha, NE 68198, USA; alanders@unmc.edu (A.A.-B.); nick.kocmich@unmc.edu (N.K.); amy.rezac@unmc.edu (A.R.-E.); 2 Department of Paediatrics, University of Abuja Teaching Hospital, Abuja 900211, Nigeria; oeyinade@yahoo.com (E.O.); demolaanigilaje@yahoo.co.uk (E.A.); samhaamal200@gmail.com (Y.T.); 3 Department of Obstetrics and Gynaecology, University of Abuja Teaching Hospital, Abuja 900211, Nigeria; docakabago@yahoo.com; 4 Department of Medical Microbiology and Parasitology, National Hospital, Abuja 900211, Nigeria; nubwa.medugu@ifain.org; 5 Department of Biostatistics, College of Public Health, University of Nebraska Medical Center, Omaha, NE 68198, USA; elyden@unmc.edu; 6 Department of Biomedical and Molecular Sciences, Queen’s University, Kinston, ON K7L 3N6, Canada; martin.kaufmann@queensu.ca (M.K.); gj1@queensu.ca (G.J.); 7 International Foundation Against Infectious Disease in Nigeria (IFAIN), Abuja 900211, Nigeria; theresa.ajose@ifain.org (T.A.); grace.olanipekun@ifain.org (G.O.); 8 Department of Pediatrics, Heersink School of Medicine, University of Alabama Birmingham, Birmingham, AL 35233, USA; sobaro@uabmc.edu; 9 Department of Medical Nutrition, College of Allied Health Professions, University of Nebraska Medical Center, Omaha, NE 68198, USA; ckhanson@unmc.edu

**Keywords:** vitamin D metabolites, newborn sepsis, newborn anthropometrics, 3-epi-25(OH)D_3_

## Abstract

Low levels of vitamin D in maternal and cord blood have been associated with neonatal sepsis. This study assessed the association of vitamin D metabolites (25(OH)D, 3-epi-25(OH)D_3_, and 24,25(OH)_2_D_3_) levels in maternal and cord blood with newborn sepsis evaluation in Nigerian mother–infant dyads. Maternal and cord blood from 534 mothers and 536 newborns were processed using liquid chromatography-tandem mass spectrometry. Spearman correlation was used to compare continuous variables, Mann–Whitney for dichotomous variables, and Kruskal–Wallis for two or more groups. High cord percent 3-epi-25(OH)D_3_ levels were positively associated with newborn evaluation for sepsis (*p* = 0.036), while maternal and cord 25(OH)D and 24,25(OH)_2_D_3_ levels were not. Being employed was positively associated with maternal and newborn 3-epi-25(OH)D_3_ concentrations (*p* = 0.007 and *p* = 0.005, respectively). The maternal 3-epi-25(OH)D_3_ and percent 3-epi-25(OH)D_3_ were positively associated with vaginal delivery (*p* = 0.013 and *p* = 0.012, respectively). Having a weight-for-age Z-score ≤ −2 was positively associated with newborn percent 3-epi-25(OH)D_3_ levels (*p* = 0.004), while a weight-for-length Z-score ≤ −3 was positively associated with maternal and newborn percent 3-epi-25(OH)D_3_ levels (*p* = 0.044 and *p* = 0.022, respectively). Our study highlights the need to further investigate the biological role of 3-epi-25(OH)D_3_ and its clinical significance in fetal growth and newborn outcome.

## 1. Introduction

Nigeria accounts for nearly one-quarter of Africa’s newborn deaths (death in the first 28 days of life) and has the 8th highest neonatal mortality rate in the world at 35 per 1000 live births [1]. A third of newborn deaths are due to infections. Exploring different approaches to reduce infection-related neonatal morbidity and mortality is crucial in the region. While mitigating programs such as maternal vaccinations [2] and screening for vertically transmitted infections [3] are important, concomitantly addressing other factors such as maternal nutrition and micronutrient status can help improve newborn health and increase survival. One such micronutrient, vitamin D, has been studied for its key role in bone metabolism and calcium homeostasis, but it also has important extra-skeletal functions as an innate and adaptive immune response modulator [4].

### 1.1. Vitamin D Physiology

Sunlight, diet, and supplements are major sources of vitamin D, which comes in two major forms: vitamin D2, 25(OH)D_2_, and vitamin D3, 25(OH)D_3_ [4]. While 25(OH)D_2_ is synthesized from plant ergosterol, mostly mushroom and yeast, 25(OH)D_3_ is synthesized in the skin by the reaction of 7-dehydrocholesterol with UVB radiation and is also found in selected animal products such as fatty fish and fish oil. Once vitamin D enters the circulation (as 25(OH)D_2_, 25(OH)D_3_, or both), it is transported to the liver bound to the vitamin D-binding protein (VDBP), where it is metabolized into 25(OH)D by the vitamin D-25-hydroxylase (CYP2R1) [5,6]. 25(OH)D is the main circulating form of vitamin D and is a biomarker of vitamin D status per the Institute of Medicine (IOM) [7]. 25(OH)D, bound to the VDBP, is then transported to the kidney, where it is metabolized by the enzyme 25-hydroxyvitamin D-1α-hydroxylase (CYP27B1) to its biologically active form, 1,25(OH)_2_D (calcitriol) [4]. The enzyme CYP27B1 is expressed in many tissues, such as activated macrophages, parathyroid glands, and the colon, for example. When 1,25(OH)_2_D is formed in these tissues, it exerts its autocrine and paracrine role by binding to the vitamin D receptor (VDR) in the nucleus to regulate different gene expressions [8,9]. Additionally, 1,25(OH)_2_D regulates its own metabolism by feedback mechanisms using the CYP24A1 gene to convert 1,25(OH)_2_D into 1,24,25(OH)_3_D as well as the conversion of 25(OH)D into 24,25(OH)_2_D [9].

During pregnancy, 25(OH)D freely crosses the placenta and is the main source of circulating vitamin D for the developing embryo; typically, cord blood 25(OH)D levels are approximately 50–75% of the maternal levels. [10,11] Vitamin D metabolism yields the previously described metabolites during pregnancy and in the newborn period; however, the functionality of the CYP24A1 enzyme is reduced, thereby suppressing the metabolism of 25(OH)D [12]. Both 25(OH)D_3_ and 25(OH)D_2_ can be alternatively metabolized through a C-3 epimerization pathway that parallels the standard metabolic pathway [13]. The concentration of the product of this epimerization process, 3-epi-25(OH)D_3_, increases at the end of the pregnancy and decreases over the first year of life of the offspring, and the presence of this metabolite can affect the accurate measurement of maternal and neonatal vitamin D status [8,9]. Singh et al. reported that in a group of 172 infants <1 year of age with detectable 25(OH)D_2_ or 25(OH)D_3_, 22.7% had C3 epimers that ranged between 8.7% and 61.1% of the total 25(OH)D, leading to an overestimation of 25(OH)D levels, and the epimer percentage was inversely correlated to the infants age (r = 0.48; *p* < 0.002) [14,15,16]. Therefore, in infants <1 year of age, an assay that allows accurate detection of 25(OH)D in the presence of its C-3 epimers, such as the liquid chromatography-tandem mass spectrometry (LC-MS/MS) method, should be used [17].

### 1.2. Effects of Vitamin D on the Immune System and Infections

In the innate immune system, when an infection occurs, activated macrophages and monocytes strongly express the enzyme CYP27B1, which then converts 25(OH)D into its active form, 1,25(OH)_2_D. This conversion further increases the antimicrobial properties of the activated macrophages and monocytes, leading to an enhancement of their antimicrobial activities, and helping them stimulate the production of an endogenous antimicrobial, cathelicidin, that can act against invasive bacteria, fungi, and viruses [17]. In the adaptive immune system, dendritic cells and activated T cells express the enzyme CPY27B1. Therefore 25(OH)D can exert an effect on them when it is converted to its active form, 1,25(OH)_2_D, by modulating immune activation and inflammation [17].

Low maternal and newborn 25(OH)D levels have been associated with an increased susceptibility to neonatal infections [18]. Several studies have shown that 25(OH)D deficiency at birth was associated with an increased risk of lower respiratory tract infections [10,11]. In a 2021 Nigerian study, a low vitamin D level in children aged 1–59 months was associated with an increased risk of acute pneumonia [19]. In addition to increased susceptibility to infections, studies have shown an association between maternal vitamin D deficiency and pregnancy complications such as intrauterine growth restriction, increased caesarian sections, and preterm birth [20]. Maternal risk factors that have been associated with maternal 25(OH)D deficiencies include the type of maternal employment and educational level [21]. Studies from low, middle, and high-income countries have shown that lack of a college education was associated with a higher risk of 25(OH)D deficiency in the mother [22].

### 1.3. Vitamin D Metabolites and Associated Outcomes

The prevalence of 25(OH)D deficiency in African settings can vary extensively based on the population, study design, and methods; in a systematic review and meta-analysis on the prevalence of vitamin D deficiency in Africa by Mogire et al., the authors found that the mean serum 25(OH)D concentrations were lower in populations living in northern African countries or South Africa compared with other African regions [17]. Furthermore, the study found that urban areas had low 25(OH)D levels compared with rural areas, women compared with men, and newborns compared with their mothers [17]. While there was sunlight almost 12 h a day, most people were dark-skinned, and often, women extensively practiced head covering for religious reasons [17]. This may have led to decreased sun exposure and a higher risk for 25(OH)D deficiency, which is of increased concern in pregnant women. The following studies conducted on pregnant women in Nigeria have looked at the prevalence of 25(OH)D deficiency in pregnancy. A 2018 study by Owie et al. showed that though the prevalence of maternal 25(OH)D deficiency was 4.8%, the associated newborn 25(OH)D deficiency was 29.5% [23]. A 2019 study by Oluwole showed that the prevalence of serum 25(OH)D deficiency was higher among women with preterm delivery than among those with term delivery [24]. No study, however, has assessed the impact of 25(OH)D and associated metabolites status on pregnancy and newborn outcomes in Nigeria [20,25,26]. No studies with the Nigerian populations have looked at associations of maternal-infant vitamin D and metabolite levels with the above risk factors and outcomes.

In contrast to 25(OH)D, 3-epi-25(OH)D_3_ does not efficiently transfer across the placenta; high levels observed in infants are likely due to post-natal epimerization of 25(OH)D [8,27]. Furthermore, 3-epi-25(OH)D_3_ has a lower affinity for the VDR than 25(OH)D; this could mean that its biological activity may be lower; this is important, as 25(OH)D may be overestimated if the assay used does not separate 3-epi-25(OH)D_3_ when measuring 25(OH)D status [28]. Though its affinity for the VDR may be lower, 3-epi-25(OH)D_3_ is reported as potent of a suppressor of parathyroid hormone (PTH) as the biologically active 1,25-(OH)_2_D [29]. Furthermore, 3-epi-25(OH)D_3_ has also been reported to possess significant activity in stimulating surfactant phospholipid synthesis in alveolar type II cells [30]. Another metabolite, 24,25(OH)_2_D_3_, the first metabolite in the process of inactivation of 25(OH)D, is thought to help stimulate growth plate development, bone or cartilage mineralization, bone fracture repair, or suppression of PTH. The effect of 3-epi-25(OH)D_3_ and 24,25(OH)_2_D_3_ on suppressing PTH is particularly important for premature infants who are at higher risk of osteopenic bone disease [4,28]. There are no reports of these metabolites being associated with the risk of sepsis in the newborn, as seen with 25(OH)D. Additionally, there are no reported studies looking at these metabolites in Nigerian mother–infant dyads.

The primary objective of this study was to assess the association of newborn sepsis evaluation to maternal and cord 25(OH)D, 3-epi-25(OH)D_3_, and 24,25(OH)_2_D_3_ levels in a population of pregnant Nigerian women and their offspring. The secondary objective was to look at associated maternal risk factors and newborn outcomes based on these maternal and cord metabolite levels.

## 2. Materials and Methods

### 2.1. Recruitment

This pilot study was conducted within a larger observational cohort study on vertically transmitted infections and neonatal outcomes that was being conducted at the Universit of Abuja Teaching Hospital (UATH) from April 2016 to March 2018. UATH serves a peri-urban settlement located in Gwagwalada, Nigeria, near Abuja, the Federal Capital Territory. A total of 2586 pregnant women ≥18 years of age were enrolled in the large observational cohort over the course of the study, after informed consent, along with their offspring, which accounted for 1033 newborn males and 963 newborn females. The samples analyzed in our pilot study were collected at the time of delivery at UATH between April 2016 and February 2017 and represented 534 mothers and 536 newborns. Reasons for not obtaining a sample were not always documented, but when information was reported, most of the reasons were that the delivery occurred at another healthcare facility/home and that, due to the timing of delivery, there were no available research personnel.

### 2.2. Ethical Approval

Ethical approval for the study was granted by the ethics committee at UATH. Additionally, a separate IRB approval was obtained at the University of Nebraska Medical Center (UNMC) and Queen’s University, given some of the investigators and the grant funding came from that institution.

### 2.3. Sample and Data Collection

A sample of 2.5 mL of maternal blood and a sample of 2.5 mL of cord blood were collected at the time of delivery from each mother who consented to participate in the study along with their newborn. Inclusion criteria were maternal age ≥18 years and newborn gestational age ≥24 weeks, and infant age at the time of enrollment could be between 0 and 7 days, though all mother and infant dyads were enrolled at or right before delivery. Additional inclusion criteria were an absence of heavy peripartal vaginal bleeding and the ability to provide written informed consent, and no specific exclusion criteria were listed for the study. Maternal infant demographic and clinical data were prospectively collected and entered in UNMC’s research electronic data capture tool termed (REDcap). Clinical data collected included maternal age, body mass index (BMI) at the time of delivery, hemoglobin, gestational age, birth anthropometrics, gender, and Apgar scores (1 min and 5 min). Infant clinical outcome data collected included 7-day and 28-day follow-up phone interviews conducted with participating mothers to assess infant well-being, interval illness, or hospitalization for newborn sepsis evaluation. Neonatal sepsis, a leading cause of morbidity and mortality, was defined as a bloodstream infection of infants in the first 28 days of life [31]. In our study, we also looked at early neonatal death (birth to <7 days of age) and late newborn death (>7 to 28 days) [32].

### 2.4. Biochemical Analysis

All blood samples were protected from direct exposure to sunlight by placing them in a brown bag after collection and then stored at −80 °C. They were batched and shipped every 3 months to UNMC on dry ice. From there, all samples were then stored again at −80 °C and shipped to Queens University, Ontario, Canada, where concentrations of 25(OH)D_3_, 25(OH)D_2_, 3-epi-25(OH)D_3_, and 24,25(OH)_2_D_3_ were measured using the liquid chromatography-tandem mass spectrometry (LC-MS/MS)-based method involving derivatization with DMEQ-TAD29 [33]. 25(OH)D was calculated by adding 25(OH)D_3_ and 25(OH)D_2_. The percent 3-epi-25(OH)D_3_ was calculated as the ratio of 3-epi-25(OH)D_3_ to 25(OH)D_3_. The Queen’s University Vitamin D Laboratory LC-MS/MS method had been accredited by DEQAS for the last 3 annual cycles at the time of the study.

### 2.5. Clinical Outcome

On days 7 and 28 of life, study personnel contacted mothers via phone interview to assess whether the infant was alive and if they had been hospitalized and evaluated for sepsis since birth. All mothers were encouraged to bring their newborns to UATH if they were ill, as they would be assessed for sepsis by screening for bacteremia and meningitis as clinically indicated. The primary endpoint for statistical analysis was the association of neonatal sepsis evaluation by day 28 of life with maternal and newborn 25(OH)D and metabolite levels.

### 2.6. Growth Outcome

Using international standards from the INTERGROWTH-21st Project, birth anthropometric parameters were measured for infant birth weight, length, and head circumference and reported in percentiles [34]. The World Health Organization (WHO) Child Growth Parameter’s Anthro software for SPSS (SPSS 29.0 Inc., Chicago, IL, USA) was used to construct the birth growth parameters z-scores. Z-scores are used to indicate the standard deviation from the mean of growth parameters (weight, height, and head circumference) when plotted by age; they are related to percentile.

### 2.7. Statistical Analysis

Data were summarized using descriptive statistics to include mean, standard deviation, median, minimum, and maximum for continuous variables, while counts and percentages were used to display categorical data. Maternal and newborn 25(OH)D levels were categorized as deficient (<20 ng/mL), insufficient (20–29 ng/mL), or sufficient (≥30 ng/mL) based on the Endocrine Society guidelines [35]. Spearman correlation coefficients were calculated to look at the association of mother and cord serum measurements and serum measurement with continuous participant characteristics. The Mann–Whitney test was used to compare median serum levels between dichotomous participant characteristics. The Kruskal–Wallis (KW) test was used to compare the median serum levels between types of maternal head covering. If the KW test was significant, pairwise comparisons for head covering distribution were adjusted using Dunn’s test. Continuous vitamin D and metabolite values were also analyzed to look for correlations with maternal age, body mass index, gestational age, and newborn anthropometrics using Spearman correlation coefficients. Logistic regression was used to adjust for maternal age as a potential confounder. Statistical significance was set at *p* ≤ 0.05. SAS version 9.3 (SAS Institute, Cary, NC, USA) was used for all analyses.

## 3. Results

A total of 534 mothers and 536 infants were included in the study. Vitamin D analysis was completed in 525 maternal and 526 cord samples available. The baseline clinical characteristics of the mothers are listed in Table 1 and the newborns are listed in Table 2. Maternal age ranges from 18–45 years with a mean age of 30 ± 5 years. The mean newborn gestational age is 38.5 ± 2.2 weeks. During the newborn period, 34 of 526 babies were evaluated for sepsis, with 10 newborns lost to follow-up. Of the 536 newborns enrolled in this study, we have survival data for 464 in the first 28 days of life; a total of 5 newborns died within the first 7 days of life, and 4 babies between days 8 and 28 of life.

### 3.1. Vitamin D Metabolites and Associated Newborn Outcomes

The association between maternal and newborn 25(OH)D and metabolite level and newborn outcomes are listed in Table 3. Maternal and infant median levels of 25(OH)D_,_ 24,25(OH)_2_D_3_, and 3-epi-25(OH)D_3_ are not significantly associated with newborn sepsis evaluation. However, the median values of percent 3-epi-25(OH)D_3_ are higher in the neonates who were evaluated for sepsis than in those who were not, and this difference is statistically significant (6.18 vs. 5.68, *p* = 0.036). Using logistic regression, cord 3-epi-25(OH)D_3_ levels remain significantly associated with sepsis evaluation after adjusting for maternal age (*p* = 0.0053). The median 25(OH)D, 24,25(OH)_2_D_3_, and 3-epi-25(OH)D_3_ levels of infants who were not alive at 28 days from birth are not significantly lower when compared with the levels in infants who were alive at 28 days.

There is no significant difference between the median maternal and infant levels of 3-epi-25(OH)D_3_ or percent 3-epi-25(OH)D_3_ with weight-for-age Z-score ≤ −2 and weight-for-length Z-score. However, newborn median percent 3-epi-25(OH)D_3_ is associated with weight-for-age Z-score ≤ −2 (6.17 vs. 5.65, *p* = 0.004) while both maternal and infant median percent 3-epi-25(OH)D_3_ are associated with weight-for-length Z-score ≤ −3 (3.76 vs. 3.58, *p* = 0.044 and 6.25 vs. 5.64, *p* = 0.022, respectively).

### 3.2. Vitamin D Metabolites and Associated Maternal Risk Factors and Outcomes

The association between maternal and newborn 25(OH)D and metabolites level and maternal risk factors, employment status, education level, outcomes, and mode of delivery are listed in Table 4. Mothers who were employed have a higher median maternal 25(OH)D level than mothers who were unemployed, and the difference is significant (37.65 ng/mL vs. 34.97 ng/mL, *p* = 0.041). Mothers who were employed have a higher median newborn 25(OH)D level than mothers who were unemployed, and the difference is also significant (24.56 ng/mL vs. 23.04 ng/mL, *p* = 0.01).

There is no evidence of a difference between the infant median concentrations of 3-epi-25(OH)D_3_ and the median percent 3-epi-25(OH)D_3_ with maternal employment status; however, mothers who were employed have higher median maternal and newborn 3-epi-25(OH)D_3_ than mothers who were unemployed, and the difference is statistically significant (1.24 ng/mL vs. 1.19 ng/mL, *p* = 0.007 and 1.37 ng/mL vs. 1.26 ng/mL, *p* = 0.005, respectively). Overall, the only significant association observed for 24,25(OH)_2_D_3_ concentration with maternal risk factors is that the median infant 24,25(OH)_2_D_3_ levels are higher for infants of mothers who are employed compared with the mothers who were not (1.06 vs. 0.98, *p* = 0.03).

Mothers with a grade 1–12 educational level have higher median maternal and newborn 3-epi-25(OH)D_3_ levels than mothers who had a tertiary level of education (1.4 ng/mL vs. 1.21 ng/mL, *p* < 0.0001 and 1.41 ng/mL vs. 1.26 ng/mL, *p* < 0.001, respectively). Mothers with a grade 1–12 educational level have higher median maternal and newborn percent 3-epi-25(OH)D_3_ levels than mothers who had a tertiary level of education, and the difference is significant. (3.76 vs. 3.5, *p* < 0.0001, and 6.29 vs. 5.39, *p* < 0.0001, respectively). Mothers who had a vaginal delivery have higher median maternal 3-epi-25(OH)D_3_ and median percent 3-epi-25(OH)D_3_ than mothers who had cesarean section, and the difference is statistically significant. (1.16 ng/mL, *p* = 0.013 and 3.68, *p* = 0.012, respectively).

### 3.3. Comparison of Mother–Infant Dyad Categories of 25(OH)D Levels

**Figure 1:**
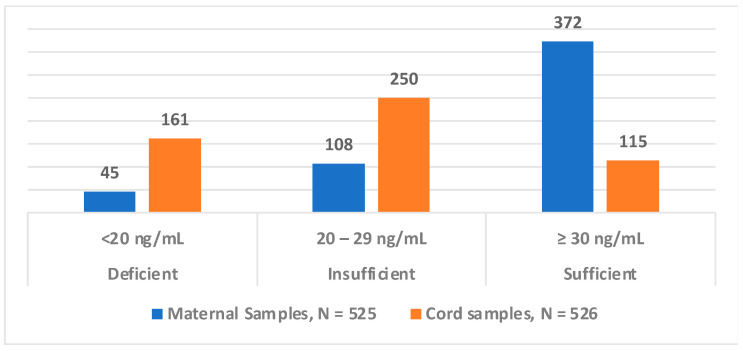
Maternal and newborn 25(OH)D categorized levels. Levels are categorized as deficient, insufficient, or sufficient based on the Endocrine Society guidelines [35]. Data show a significant difference between maternal and cord blood (*p* < 0.001).

Most mothers, 70.9% (372/525), had sufficient levels of 25(OH)D, while only 21.9% (115/526) of the newborns had sufficient levels. Most newborns had insufficient levels at 47.5% (250/526). There is a significant difference in the categorized concentrations of 25(OH)D levels between maternal and cord blood levels (*p* < 0.001) (Figure 1).

### 3.4. Type of Maternal Head Covering and 25(OH)D Status in Mother–Infant Dyads

**Figure 2:**
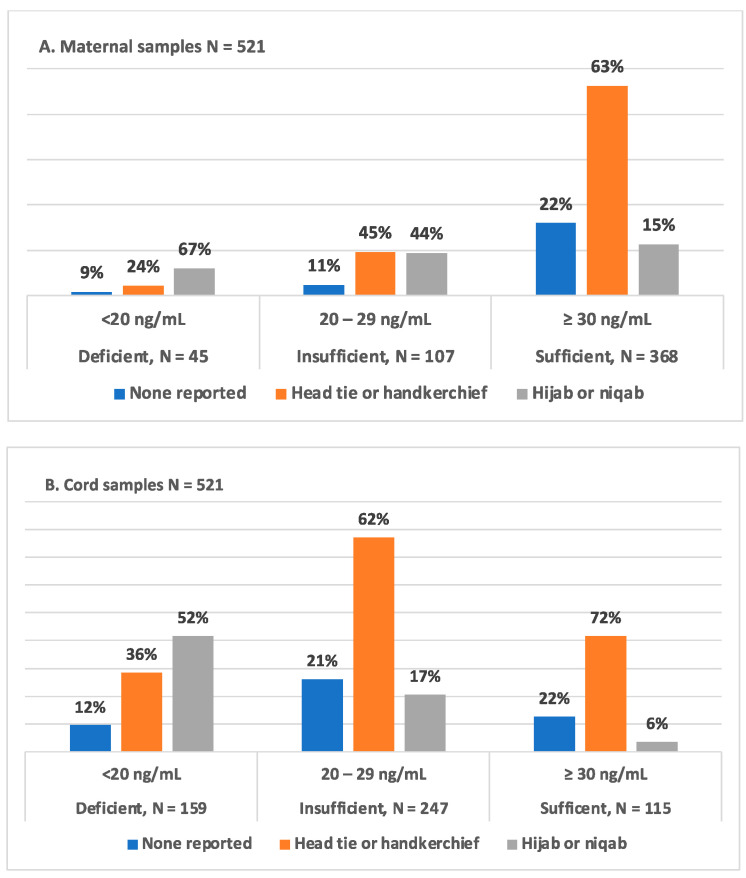
Head covering versus maternal and cord 25(OH)D category levels. Comparing the type of head covering used to maternal and cord vitamin D levels. (**A**) There is a significant difference in the median maternal 25(OH)D levels between mothers who used a head tie versus a hijab and between mothers who used no head covering versus wearing a hijab (*p* < 0.0001). (**B**) There is a significant difference in median cord 25(OH)D levels between mothers who used a head tie versus a hijab and between mothers who used no head covering versus wearing a hijab (*p* < 0.0001).

Figure 2 shows the association of type head covering by maternal and infant 25(OH)D levels. In Figure 2A, most mothers with sufficient levels of 25(OH)D wore a head tie 62.7% (231/368), while most mothers with deficient 25(OH)D levels wore a hijab 66.6% (30/45). In Figure 2B, most infants who have deficient levels of 25(OH)D had mothers who wore a hijab 52.2% (83/159), while most infants who have insufficient levels of 25(OH)D wore a head tie 62.3% (154/247). There is a difference in the median maternal and cord 25(OH)D, 3-epi-25(OH)D_3_, and 24,25(OH)_2_D_3_ levels when comparing mothers who wore a head tie versus a hijab and those who wore no head garment versus a hijab, with *p* < 0.0001 for both pairwise combinations.

### 3.5. Correlations Associated with Mother–Infant Dyad 25(OH)D and Metabolite Levels

The correlation of the mean maternal–newborn 25(OH)D metabolite levels is listed in Table 5. The mean maternal 25(OH)D level at 36.72 (±11.6) ng/mL is higher than the mean newborn 25(OH)D level at 24.52 (±8.14) ng/mL. The mean 3-epi-25(OH)D_3_ in maternal samples at 1.33 (±0.61) ng/mL accounts for 3.8% (±1.14) of the total 25(OH)D concentration. In cord blood samples, absolute levels of 3-epi-25(OH)D_3_ are like maternal levels at 1.39 (±0.59) ng/mL, but the percentage of 25(OH)D comprised by 3-epi-25(OH)D_3_ is higher at 5.9% (±1.6). Mothers have higher 24,25(OH)_2_D_3_ means at 1.77 (±0.9) compared with infants who are at 1.09 (±0.57). Maternal and cord 25(OH)D and 24,25(OH)_2_D_3_ levels are positively correlated (r = 0.7, *p* < 0.0001; r = 0.81, *p* < 0.0001). Concentrations and the percentage of 3-epi-25(OH)D_3_ in maternal and cord samples are positively correlated as well (r = 0.84, *p* < 0.0001; r = 0.66, *p* < 0.0001, respectively).

A correlation of continuous mother and infant characteristics with 25(OH)D, 24,25(OH)_2_D_3_ and 3-epi-25(OH)D_3_ levels and percent 3-epi 25(OH)D_3_ show a positive association between maternal age with cord 24,25(OH) _2_D_3_ levels (r = 0.1, *p* = 0.03) and gestational age and cord percent 3-epi 25(OH)D_3_ (r = −0.1, *p* = 0.02). Birthweight is positively associated with maternal 25(OH)D levels and cord 24,25(OH) _2_D_3_ levels (r = 0.1 and *p* = 0.03 and r = 0.11 and *p* = 0.01, respectively), while it is negatively associated with cord 25(OH)D levels (r = −0.12 and *p* = 0.005). There is no other significant correlation between 25(OH)D levels with maternal age, BMI, gestational age, hemoglobin, birth weight, birth length, and head circumference.

## 4. Discussion

This study is the first to evaluate 25(OH)D, 3-epi 25(OH)D_3_, 24,25(OH) _2_D_3_, and associated risk factors and pregnancy outcomes in a population of Nigerian women and their newborn at the time of delivery using the highly sensitive LC-MS/MS method.

### 4.1. Primary Outcome

Our study showed that high cord median percent 3-epi-25(OH)D_3_ was significantly associated with being evaluated for newborn sepsis (*p* = 0.036). Maternal median 3-epi-25(OH)D_3_ and percent 3-epi-25(OH)D_3_, as well as cord median 3-epi-25(OH)D_3_, were not associated with sepsis evaluation. Median maternal and cord 25(OH)D and 24,25(OH)_2_D_3_ were also not associated with sepsis evaluation. There was also no significant association between the maternal and cord levels of these three metabolites and newborn death within the first 28 days of life.

A 2021 systematic review and meta-analysis of 18 studies by Workneh Bitew et al. reported that low levels of 25(OH)D in maternal and cord blood were associated with newborn sepsis [18]. A 2022 meta-analysis of 42 randomized controlled trials by Liu et al. showed that vitamin D supplementation during pregnancy was associated with a lower risk of intrauterine or neonatal death (RR, 0.69; 95% CI, 0.48–0.99) [36]. The lack of a significant association between 25(OH)D levels and newborn sepsis evaluation and newborn death could be due to the low incidence of these events in our study population compared with the larger trials described by Workneh Bitew et al. and Liu et al.

A review of the literature did not reveal other studies that have investigated the association of 3-epi-25(OH)D_3_, percent 3-epi-25(OH)D_3_, or 24,25(OH)_2_D_3_ with newborn sepsis. Percent 3-epi-25(OH)D_3_ represents the percentage of 25(OH)D that is in the epimer form [37]. This means a higher 3-epi-25(OH)D_3_ indicates more circulating 3-epi-25(OH)D_3_ and lower circulating 25(OH)D. Our findings of an association between the evaluation for newborn sepsis and high 3-epi-25(OH)D_3_ may be associated with these low 25(OH)D stores in the newborn, which is known to predispose newborns to adverse outcomes, including sepsis.

### 4.2. Vitamin D and Metabolite Status

Maternal and cord 25(OH)D, 3-epi 25(OH)D_3_, and 24,25(OH)_2_D_3_ levels were all positively correlated (r = 0.7, r = 0.84, and r = 0.81, respectively, with *p* < 0.0001 for all). This is consistent with what has been previously described [38,39]. Most mothers, 70.86%, had sufficient levels of 25(OH)D. In contrast, only 21.9% of the newborns had sufficient levels of 25(OH)D. The prevalence of maternal 25(OH)D deficiency and insufficiency was 8.6% and 20.6%, respectively. The prevalence of neonatal 25(OH)D deficiency and insufficiency was 30.6% and 47.5%, respectively. These observations suggest that there may not be an efficient transfer of 25(OH)D to the newborn or that there is an independent regulation of 25(OH)D in the mother and the newborn [38,39]. These observations between sufficient maternal 25(OH)D levels and deficient newborn 25(OH)D levels have been reported in several studies [40,41].

The cord 25(OH)D mean concentrations observed in our study were similar to those reported in a Lagos study by Owie et al., where the prevalence of newborn 25(OH)D deficiency and insufficiency were 28.3% and 46.1%, respectively [23]. In the same study, the prevalence of maternal 25(OH)D deficiency was about half our values at 4.8%, while the prevalence of insufficient 25(OH)D levels was about 50% higher. Lagos is a large metropolitan area, while UATH serves a more peri-urban and rural settlement; the differences in geographic settings would not clearly explain the varying results. Furthermore, it would also seem that even with the differences in maternal stores of 25(OH)D in both study populations, a similar distribution of newborns with 25(OH)D deficient and insufficient levels was noted. Owie et al. used an enzyme-linked immunosorbent assay (ELISA) method to assess vitamin D, while we used an LC-MS/MS assay. The prevalence of deficient and insufficient 25(OH)D levels in the newborn population may be higher in the Owie study if the measurement of 25(OH)D included the concentration of 3-epi-25(OH)D_3_ based on their study assay; we avoided this problem by using the LC-MS/MS method. In a study by Oluwole et al. also conducted in Lagos, the authors reported that 14.1% of the mothers had deficient vitamin D levels. However, the authors defined deficient at levels of <30 ng/mL. We defined deficient as <20 ng/mL and insufficient 20–29 ng/mL per the Endocrine Society guidelines [35]. If we had used a similar cut-off as the Oluwole team, our prevalence of deficient levels would have increased to 29.2%, which would then reflect a higher prevalence of 25(OH)D deficiency [42]. In the Oluwole study, newborn levels were not assessed. In another study in Lagos by Gbadegesin et al., maternal vitamin D levels were only collected at an antenatal visit between gestational ages 10 and 28 weeks. The authors reported a prevalence of vitamin D deficiency and insufficiency of 29% and 10.4%, which are higher than what we observed in our study. Gbadegesin et al. did not repeat maternal serum sampling at delivery. We, however, collected samples at delivery, and most deliveries were term, with a mean gestational age of 38.4. Moreover, knowing that vitamin D levels progressively decline with pregnancy, the prevalence of vitamin deficiency and insufficiency in the Gbadegesin et al. study might be higher. [23,24] To have more reliable data on the prevalence of vitamin D deficiency and insufficiency in Nigeria, it would be important to have reference levels at different stages of a pregnancy and that consistent specific guidelines for 25(OH)D level categorization be followed.

We also measured the metabolites 3-epi-25(OH)D_3_ and 24,25(OH) _2_D_3_ in our study; normal levels for these metabolites have not yet been determined. With regards to 3-epi-25(OH)D_3_, however, distinguishing between 25(OH)D and 3-epi 25(OH)D_3_is of biological relevance, particularly in infants; 3-epi-25(OH)D_3_ accounts for a significant proportion of the circulating total 25(OH)D and increases at the end of the pregnancy and decreases over the first year of life [20]. Access to an adequate assay method such as LC-MS/MS can help provide an accurate measurement. In our study, the 3-epi-25(OH)D_3_ and percent 3-epi-25(OH)D_3_ in the newborn were generally higher than the maternal 3-epi-25(OH)D_3_ and percent 3-epi-25(OH)D_3_ except for the infant who was not alive at 28 days from birth where the newborn values were lower for both 3-epi-25(OH)D_3_ and percent 3-epi-25(OH)D_3_ than for the mothers. These data suggest that the fetus may contribute significantly to 3-epi-25(OH)D_3_ production. Some studies have suggested that 3-epi-25(OH)D_3_ may not transfer efficiently across the placenta and that it may be generated endogenously by the fetus from maternal 25(OH)D stores [14,15,16]. Other studies have shown that after birth, 3-epi-25(OH)D_3_ may be generated from exogenous sources such as specific C3 epimerized vitamin D_3_ supplements [27,28]. A randomized controlled trial of premature infants receiving specifically 25 (OH)D_3_ supplementation showed an increase of their 3-epi-25(OH)D_3_ from 6–8% of total 25(OH)D_3_ to 30–45% of total 25(OH)D_3_ after 4 and 8 weeks of 25(OH)D_3_ supplementation [43]. In this study, 25(OH)D2 levels were insignificant, so serum 25(OH)D_3_ instead of 25(OH)D was used to assess vitamin D status. An epimerization pathway only active in the first year of life has also been suggested as a possible way of endogenous production of 3-epi-25(OH)D_3_ [14,15,16].

With regards to 24,25(OH)_2_D_3_, the metabolism of 25(OH)D into 24,25(OH)_2_D_3_ decreases in pregnancy to maintain persistently elevated serum 1,25(OH)_2_D, the biologically active form of 25(OH)D [44]. Data from non-pregnant populations suggest that serum 24,25(OH)_2_D_3_ and the ratio of serum 24,25(OH)_2_D_3_ to 25(OH)D are useful indicators of 25(OH)D deficiency, and they have been reported to help predict the response to 25(OH)D supplementation [43]. In the previously reported randomized trial by Hanson et al., premature infants who received 25(OH)D_3_ supplementation had an increase in 24,25(OH)_2_D_3_ values over time, and there was a high correlation between concentrations of 25(OH)D_3_ and 24,25(OH)_2_D_3_. Furthermore, the same study observed a positive association between the ratio of 25(OH)D_3_:24,25(OH)_2_D_3_ and PTH concentrations (r = 0.52, *p* = 0.02) which was not observed at 4 weeks. This ratio has also been observed to increase linearly during times of rapid linear growth where there is an increased demand for calcium and phosphorus. The utility of these indicators during pregnancy needs further investigation [44]. In our study, we did not calculate the 25(OH)D_3_:24,25(OH)_2_D_3_ for our participants.

### 4.3. Birth Anthropometrics

Our study did not show an association of maternal or newborn 25(OH)D levels with newborn outcomes such as weight-for-age or weight-for-length. A critical review showed that although 25(OH)D is essential for fetal growth and has been associated with intrauterine growth restriction in some studies. Some investigations, however, have failed to establish any association between 25(OH)D levels and fetal birth weight [8]. Newborn higher percent 3-epi-25(OH)D_3_ levels were positively associated with weight-for-age Z-score ≤ −2 (*p* = 0.004) while both maternal and infant percent 3-epi-25(OH)D_3_ were positively associated with weight-for-length Z-score ≤ −3 (*p* = 0.044 and *p* = 0.022, respectively), these positive associations with percent 3-epi-25(OH)D_3_ for some growth parameters could be explained but the role percent 3-epi-25(OH)D_3_ calcium homeostasis and as a suppressor of PTH [45]. Finally, the median cord 24,25(OH)_2_D_3_ level was positively associated with higher birthweight (*p* = 0.01), which could be explained by the role of 24,25(OH)_2_D_3_ in helping stimulate growth plate development and bone mineralization [4,28].

### 4.4. Maternal Risk Factors and Newborn Outcomes

Maternal 25(OH)D levels decline as pregnancy advances due to progressive fetal physiological demands; our study design did not allow us to compare predelivery and delivery levels of 25(OH)D that may have affected newborn outcomes [46]. Though we did not look at maternal vitamin D supplementation like Liu et al., we also did not ask mothers if they were taking vitamin D supplements on their own. Most of our mothers, 70.86% (372/525), however, had sufficient levels of 25(OH)D based on the Endocrine Society guidelines; the mean level was 36.72 ng/mL (+/− 11.6) [35]. There are recommendations on the amount of vitamin D that should be supplemented during pregnancy, and there is evidence that supplementation early on during pregnancy leads to better newborn outcomes; the ideal maternal 25(OH)D concentration throughout pregnancy for the best outcome is not yet determined.

Luxwolda et al., in a study with Tanzanian tribes, reported that pregnant mothers had mean levels of 25(OH)D that were 60 ng/mL, while non-pregnant women had mean levels of 46 ng/mL [47]. Holles et al. used these data to develop a mathematical model that recommended circulating levels of 25(OH)D should be >40 ng/mL during pregnancy [48]. There is no consensus on what the normal pregnancy values should be during pregnancy; the IOM recommends a level of 20 ng/mL as sufficient, while the Endocrine Society guidelines recommend 30 ng/mL [35]. Our study showed that even when most mothers had sufficient 25(OH)D levels, most newborn 25(OH)D levels were in the insufficient and deficient categories. This would imply that sufficient maternal 25(OH)D stores were not enough in our study population for the newborns to achieve sufficient 25(OH)D levels as well. Studies looking at maternal 25(OH)D levels throughout pregnancy, with and without vitamin D supplementation, and collecting cord 25(OH)D levels as well could help determine what the ideal 25(OH) levels during pregnancy should be to achieve sufficient cord 25(OH)D levels and better newborn outcomes.

Our study showed significant associations between maternal 25(OH)D levels and the type of head covering used. The median 25(OH)D maternal levels between mothers who used a head tie versus a hijab and between mothers who used no head covering versus wearing a hijab (*p* < 0.0001). The median 25(OH)D cord levels between mothers who used a head tie versus a hijab and between mothers who used no head covering versus wearing a hijab (*p* < 0.0001). Most mothers who had 25(OH)D deficiency wore a hijab, while most mothers with 25(OH)D insufficiency wore a head tie. Mothers who did not use a type of head covering had higher 25(OH)D levels due to increased exposure to the sun, which may be further influenced by working outdoors versus indoors. These observations were consistent with previous reports on the effects of covering styles on 25(OH)D levels [40,41]. A higher proportion of infants born to mothers who wore a head tie or a hijab had deficient or insufficient vitamin 25(OH)D levels compared with infants of mothers who used a head covering. In our study, the percent of mothers who had sufficient levels of 25(OH)D was higher than the percent with insufficient or deficient levels, regardless of the type of head covering. This finding did not translate to a similar proportionate distribution in the newborn 25(OH)D levels. Further studies are needed in the region to better understand the multifactorial contributions of diet, dressing styles, and 25(OH)D supplementations and work to help maintain optimal maternal stores of 25(OH)D that can lead to sufficient levels for the newborn. Of note, in our study population, 25(OH)D supplementation was not part of routine prenatal care, and we did not ask mothers if they were independently taking any. Supplementation has been shown to affect maternal 25(OH)D levels in Nigerian populations, along with covering style and residing in a metropolitan versus rural area, in Owie et al. and Oluwole et al. reports [23,24].

In our study, maternal and newborn 25(OH)D levels were both significantly higher in mothers who were employed versus unemployed (*p* = 0.041 and *p* = 0.01, respectively). Kofi-Amegah et al., in a study in Ghana, looked at factors that influenced dietary and non-dietary 25(OH)D intake among pregnant women and noted that women who were employed had higher exposure to sunlight (*p* < 0.0001). Because Ghana is in geographic proximity to Nigeria, our study findings showing an association between 25(OH)D levels and employment status could also be potentially explained by sunlight exposure [42]. Owie et al. and Oluwole articles on 25(OH)D levels in pregnant women in Nigeria did not report maternal employment status in relationship to 25(OH)D levels [23,24]. El-Khateeb et al., in a study conducted in Jordan, found that low 25(OH)D levels were associated with women being unemployed (*p* < 0.001) [49]. Brian et al. found in a study of pregnant women in Nigeria that the workplace location was associated with 25(OH)D status; women who worked indoors had lower 25(OH)D levels than those who worked outdoors (*p* < 0.031) [20]. However, we did not specifically ask our study participants about the amount of sunlight exposure they had daily, nor did we inquire about the details of their jobs if they were employed.

Other significant associations were observed regarding maternal employment status: mothers who were employed had higher median maternal and newborn 3-epi-25(OH)D_3_ than unemployed mothers (*p* = 0.007 and *p* = 0.005, respectively). The median cord 24,25(OH)_2_D_3_ level was positively associated with being employed (*p* = 0.03). Given that both 3-epi-25(OH)D_3_ and 24,25(OH)_2_D_3_ are metabolites of 25(OH)D, the likelihood of higher maternal levels of 25(OH)D could lead to high levels of these substrates in a category such as employment. Working outdoors, which may be more likely in the peri-urban setting of our study population, could explain this finding. However, though we asked about employment, we did not inquire about the type of employment, which could have helped our interpretation of these findings.

Unlike other studies, we showed no association between maternal or newborn 25(OH)D levels to maternal educational level [14,15,16]. Mothers with a grade 1–12 educational level had higher median maternal and newborn 3-epi-25(OH)D_3_ than mothers who had a tertiary level of education (both with *p* < 0.0001). Some of the associations above could reflect situations where a mother is likely to have increased sun exposure based on employment status and educational level, though our study did not specifically investigate the amount of sun exposure based on employment and educational attainment.

Looking at the mode of delivery, we found no association with 25(OH)D levels. Observational reports have described an increased risk of cesarean section with vitamin D deficiency. A systematic review of 25(OH)D levels and pregnancy outcomes showed that there was an increased risk of cesarean sections in mothers with 25(OH)D deficiency and insufficiency, likely due to reduced muscle mass and strength in the pelvic floor [50]. The prevalence of cesarean sections in our study population was 25.7%, which is because our study was conducted at a referral teaching hospital that has a high caseload of complicated obstetric cases that may require cesarean section for improved maternal and/or fetal outcomes. Mothers who had a vaginal delivery had higher medial maternal 3-epi-25(OH)D_3_ and median percent 3-epi-25(OH)D_3_ than mothers who had a caesarian section (*p* = 0.013 and *p* = 0.012, respectively). While studies have shown that 3-epi-25(OH)D_3_ can be elevated in pregnancy, the reason for this mechanism is unclear, and there are no reports on an association between 3-epi-25(OH)D_3_ and mode of delivery [51]. In a 2022 study by Mao et al., where the authors looked at maternal and cord 3-epi-25-OH-D_3_ levels and pregnancy outcome, they reported that pregnancy with a male fetus, ambient solar radiation, and maternal glycemia were associated with maternal 3-epi-25-OH-D_3_ levels [8]. When examining the cord blood 3-epi-25(OH)D_3_ levels, the study found that they were associated with higher maternal age, multiparity, maternal gestational weight gain, maternal glycemia, and earlier gestational age at delivery. Of the associations Mao et al. looked at in their research, in our study, there was a positive association between maternal age and cord 24,25(OH)_2_D_3_ levels (r = 0.1, *p* = 0.03) and gestational age and cord percent 3-epi 25(OH)D_3_ (r = −0.1, *p* = 0.02). Further studies conducted in diverse populations are needed to further explore the associations observed with 3-epi-25(OH)D_3_ and 24,25(OH)_2_D_3_.

### 4.5. Limitations

Our study had several limitations. As our study was conducted within a larger study, it was not powered to detect the association of neonatal sepsis with 25(OH)D deficiency that has been observed in a systematic review [18]. Mothers enrolled in our study came from a tertiary referral institution in a major metropolitan area of Nigeria; given that a significant number of deliveries in the region occur in the home, the population studied was not reflective of the community’s pregnant population [46]. Furthermore, the population may also not be representative of the country’s diverse diet, climate, clothing styles, and urban versus rural regions, all factors that can affect 25(OH)D levels. Finally, a lack of detailed information on specific dietary intake and 25(OH)D supplementation could have affected the levels of 25(OH)D and metabolites obtained collected from the study participants.

## 5. Conclusions

Our study is the first study to highlight a significant positive association between the cord percent 3-epi-25(OH)D_3_ with neonatal sepsis evaluation. Furthermore, 3-epi-25(OH)D_3_ and percent 3-epi-25(OH)D_3_ were associated with important maternal factors such as employment status, educational level, and mode of delivery. Newborn outcomes associated with this metabolite include weight-for-age and weight-for-length. Larger prospective studies may help better characterize the biological and clinical significance of 3-epi-25(OH)D_3_ and the percent 3-epi-25(OH)D_3_ levels in pregnant mothers and their offspring.

## Figures and Tables

**Table 1:** Maternal characteristics.

Maternal Characteristics		
Continuous variables	N	Mean (SD)
Age (years)	535	30 (5)
BMI (Kg/m^2^)	535	30 (5.34)
Hemoglobin (g/dL)	426	11.4 (1.1)
Categorical variables	N (%)	
**Education level**		
Grade 1–12	208 (47.5)	
Tertiary	309 (49.5)	
**Employed**		
Yes	341 (64.2)	
No	190 (35.8)	
**Type of head covering**		
None reported	96 (18.4)	
Hijab or niqab	131 (25.3)	
Head tie or handkerchief	294 (56.4)	
**HIV status**		
Yes	22 (4.3)	
No	487 (95.7)	
**Malaria status**		
Yes	21 (3.9)	
No	62 (11.6)	
Unknown	453 (84.5)	
**Mode of delivery**		
Vaginal	398 (74.3)	
Caeserean section	138 (25.7)	

**Table 2:** Newborn characteristics.

Newborn Characteristics		
Continuous variable	N	Mean (SD)
Gestational age (weeks)	536	38.4 (2.2)
**Birth anthroprometrics**		
Birth weight (g)	536	3088 (527.2)
Birth length (cm)	536	48.7 (4)
Birth head circumference (cm)	533	34.5 (2.8)
Categorical variables	N (%)	
**Education level**		
Grade 1–12	261 (49)	
Tertiary	275 (51)	
**Apgar at 1 min**		
>7	470 (78.8)	
≤7	48 (14.1)	
Unknown	**18 (7.1)**	
**Apgar at 5 min**		
>7	500 (94.4)	
≤7	19 (3.5)	
Unknown	17 (3.2)	
**Newborn evaluation for sepsis**		
Yes	34 (6.3)	
No	492 (91.8)	
Unknown	10 (1.9)	
**Newborn alive at 7 days**		
Yes	450 (83.9)	
No	5 (1)	
Unknown	81 (15.1)	
**Newborn alive at 28 days**		
Yes	446 (83.2)	
No	9 (1.7)	
Unknown	81 (15.1)	

**Table 3:** Newborn clinical outcomes associated with maternal and newborn vitamin D and metabolite levels.

**Maternal and newborn vitamin D and metabolites levels vs. newborn evaluation for sepsis**					
**Evaluation for sepsis**	**Yes**		**No**		
	Median	IQR	Median	IQR	*p* value
Mother (N = 525)					
25(OH)D ng/mL	32.82	15.44	37.09	16.26	*p* = 0.112
24,25(OH)_2_D_3_	1.47	0.96	1.75	1.27	*p* = 0.212
3-epi-25(OH)D_3_ ng/mL	1.24	0.72	1.28	0.69	*p* = 0.679
3-epi-25(OH)D_3_ %	3.78	1.13	3.58	1.36	*p* = 0.213
Cord (N = 526)					
25(OH)D ng/mL	22.36	7.79	24	10.29	*p* = 0.308
24,25(OH)_2_D_3_	0.96	0.65	1.03	0.77	*p* = 0.529
3-epi-25(OH)D_3_ ng/mL	1.34	0.82	1.3	0.8	*p* = 0.524
**3-epi-25(OH)D_3_ %**	**6.18**	**2.16**	**5.68**	**2.12**	***p* = 0.036**
**Maternal and newborn vitamin D and metabolites levels vs. newborn survival at 28 days**					
**Newborn survival at 28 days**	**Yes**		**No**		
	Median	IQR	Median	IQR	*p* value
Mother (N = 455)					
25(OH)D ng/mL	36.7	16.74	31.61	10.03	*p* = 0.06
24,25(OH)_2_D_3_	1.73	1.31	1.36	0.93	*p* = 0.205
3-epi-25(OH)D_3_ ng/mL	1.27	0.71	1.03	0.57	*p* = 0.212
3-epi-25(OH)D_3_ %	3.57	1.34	3.76	1.55	*p* = 0.944
Cord (N = 456)					
25(OH)D ng/mL	23.48	10.44	23.35	7.15	*p* = 0.574
24,25(OH)_2_D_3_	1.02	0.81	0.99	0.59	*p* = 0.870
3-epi-25(OH)D_3_ ng/mL	1.3	0.83	0.69	0.43	*p* = 0.377
3-epi-25(OH)D_3_ %	5.65	2.06	2.5	2.62	*p* = 0.626
**Maternal and newborn vitamin D and metabolites levels vs. weight for age Z-score ≤ −2**					
**Weight for age z-score ≤ −2**	**Yes**		**No**		
	Median	IQR	Median	IQR	*p* value
Mother (N = 524)					
25(OH)D ng/mL	33.36	14.73	37.15	16.79	*p* = 0.156
24,25(OH)_2_D_3_	1.76	1.01	1.74	1.28	*p* = 0.592
3-epi-25(OH)D_3_ ng/mL	1.17	0.7	1.29	0.7	*p* = 0.698
3-epi-25(OH)D_3_ %	3.6	1.4	3.59	1.35	*p* = 0.554
Cord (N = 525)					
25(OH)D ng/mL	22.16	7.25	24.07	10.29	*p* = 0.139
24,25(OH)_2_D_3_	0.97	0.76	1.03	0.77	*p* = 0.77
3-epi-25(OH)D_3_ ng/mL	1.3	0.83	1.31	0.79	*p* = 0.502
**3-epi-25(OH)D_3_ %**	**6.17**	**2.29**	**5.65**	**2.06**	***p* = 0.004**
**Maternal and newborn vitamin D and metabolites levels vs. weight for length Z-score ≤ −3**					
**Weight for for length Z-score ≤ −3**	**Yes**		**No**		
	Median	IQR	Median	IQR	*p* value
Mother (N = 471)					
25(OH)D ng/mL	35.75	14.84	37.55	16	*p* = 0.282
24,25(OH)_2_D_3_	1.7	1.25	1.75	1.3	*p* = 0.641
3-epi-25(OH)D_3_ ng/mL	1.42	0.87	1.28	0.65	*p* = 0.358
**3-epi-25(OH)D_3_ %**	**3.76**	**2.01**	**3.58**	**1.31**	***p* = 0.044**
Cord (N = 470)					
25(OH)D ng/mL	24.53	11.69	23.58	9.93	*p* = 0.795
24,25(OH)_2_D_3_	1.11	0.79	1.01	0.71	*p* = 0.79
3-epi-25(OH)D_3_ ng/mL	1.44	0.86	1.3	0.76	*p* = 0.192
**3-epi-25(OH)D_3_ %**	**6.25**	**2.21**	**5.64**	**1.97**	***p* = 0.022**

Note: associations with *p* < 0.05 are bolded.

**Table 4:** Maternal factors and outcomes associated with maternal and newborn vitamin D and metabolite levels.

**Maternal newborn vitamin D and metabolites status vs. employment status**					
**Employment status**	**Yes**		**No**		
	Median	IQR	Median	IQR	*p* value
Mother (N = 521)					
**25(OH)D ng/mL**	**37.65**	**15.47**	**34.97**	**17.71**	***p* = 0.041**
24,25(OH)_2_D_3_	1.74	1.18	1.74	1.42	*p* = 0.122
**3-epi-25(OH)D_3_ ng/mL**	**1.24**	**0.72**	**1.19**	**0.77**	***p* = 0.007**
3-epi-25(OH)D_3_ %	3.61	1.25	3.57	1.67	*p* = 0.2
Cord (N = 526)					
**25(OH)D ng/mL**	**24.56**	**10.29**	**23.04**	**9.84**	***p* = 0.01**
**24,25(OH)_2_** **D_3_**	**24.56**	**0.72**	**0.98**	**0.75**	***p* = 0.03**
**3-epi-25(OH)D_3_ ng/mL**	**1.37**	**0.76**	**1.26**	**0.88**	***p* = 0.005**
3-epi-25(OH)D_3_ %	5.78	2.13	5.66	2.21	*p* = 0.394
**Maternal newborn vitamin D and metabolites status vs. education leve**					
**Education level**	**Grade 1-12**		**Tertiary**		
	Median	IQR	Median	IQR	*p* value
Mother (N = 517)					
25(OH)D ng/mL	38.89	14.1	35.66	17.69	*p* = 0.07
24,25(OH)_2_D_3_	1.84	1.03	1.67	1.36	*p* = 0.092
**3-epi-25(OH)D_3_ ng/mL**	**1.4**	**0.68**	**1.21**	**0.73**	***p* < 0.0001**
**3-epi-25(OH)D_3_ %**	**3.76**	**1.52**	**3.5**	**1.24**	***p* < 0.0001**
Cord (N = 518)					
25(OH)D ng/mL	24.37	9.83	23.41	10.52	*p* = 0.332
24,25(OH)_2_D_3_	1.05	0.67	1	0.82	*p* = 0.252
**3-epi-25(OH)D_3_ ng/mL**	**1.41**	**0.76**	**1.26**	**0.79**	***p* = 0.001**
**3-epi-25(OH)D_3_ %**	**6.29**	**2.19**	**5.39**	**1.99**	***p* < 0.0001**
**Maternal newborn vitamin D and metabolites status vs. mode of delivery**					
**Mode of delivery**	**Vaginal**		**C-section**		
	Median	IQR	Median	IQR	*p* value
Mother (N = 525)					
25(OH)D ng/mL	37.01	15.31	36.44	18.11	*p* = 0.675
24,25(OH)_2_D_3_	1.77	1.26	1.59	1.27	*p* = 0.249
**3-epi-25(OH)D_3_ ng/mL**	**1.32**	**0.69**	**1.16**	**0.7**	***p* = 0.013**
**3-epi-25(OH)D_3_ %**	**3.68**	**1.46**	**3.45**	**1.07**	***p* = 0.012**
Cord (N = 526)					
25(OH)D ng/mL	23.44	10.47	24.42	8.83	*p* = 0.717
24,25(OH)_2_D_3_	1.03	36	1.02	0.67	*p* = 0.629
3-epi-25(OH)D_3_ ng/mL	1.33	0.8	1.27	0.8	*p* = 0.385
3-epi-25(OH)D_3_ %	5.77	2.19	5.63	1.97	*p* = 0.413

Note: associations with *p* < 0.05 are bolded.

**Table 5:** Correlation of the mean maternal and newborn 25(OH)D and metabolite levels.

Measurements	Mother	Newborn	r	*p* Value
	N = 526	N = 526		
25(OH)D_2_, ng/mL	2.64 ± 1.46	1.3 ± 0.57	0.91	<0.0001
25(OH)D_3_, ng/mL	34.08 ± 11.6	22.28 ± 8.03	0.7	<0.0001
25(OH)D, ng/mL	36.72 ± 11.6	24.52 ± 8.14	0.7	<0.0001
3-epi-25(OH)D_3_, ng/mL	1.33 ± 0.57	1.39 ± 0.59	0.84	<0.0001
3-epi-25(OH)D_3_ %	3.8 ± 1.14	5.9 ± 1.6	0.66	<0.0001
24,25(OH)_2_D_3_	1.77 ± 0.90	1.09 ± 0.57	0.81	<0.0001

## Data Availability

The data presented in this study are available upon request from the corresponding author. The data are not publicly available due to privacy reasons.
